# Barriers to following imaging guidelines for the treatment and management of patients with low-back pain in primary care: a qualitative assessment guided by the Theoretical Domains Framework

**DOI:** 10.1186/s12875-022-01751-6

**Published:** 2022-06-03

**Authors:** Andrea Pike, Andrea Patey, Rebecca Lawrence, Kris Aubrey-Bassler, Jeremy Grimshaw, Sameh Mortazhejri, Shawn Dowling, Yamile Jasaui, De-implementing Wisely Research Group, Sacha Bhatia, D’Arcy Duquettes, Erin Gionet, Kyle Kirkham, Wendy Levinson, Brian Johnston, Kelly Mrklas, Patrick Parfrey, Justin Presseau, Todd Sikorski, Monica Taljaard, Kednapa Thavorn, Krista Mahoney, Shannon Ruzycki, Amanda Hall

**Affiliations:** 1 Primary Healthcare Research Unit, Faculty of Medicine, Health Sciences Centre, Memorial University, Rm 421, Janeway Hostel, 300 Prince Phillip Drive, St. John’s, NL A1B 3V6 Canada; 2 Centre for Implementation Research, Ontario Health Research Institute, Ottawa, ON Canada; 3 University of Calgary, Calgary, AB Canada

**Keywords:** Low back pain, Clinical practice guidelines, Imaging, De-implementation, Low-value care, x-ray, CT, Evidence-based medicine, Theoretical domains framework, TDF

## Abstract

**Background:**

Low back pain (LBP) is a leading cause of disability and is among the top five reasons that patients visit their family doctors. Over-imaging for non-specific low back pain remains a problem in primary care. To inform a larger study to develop and evaluate a theory-based intervention to reduce inappropriate imaging, we completed an assessment of the barriers and facilitators to reducing unnecessary imaging for NSLBP among family doctors in Newfoundland and Labrador (NL).

**Methods:**

This was an exploratory, qualitative study describing family doctors’ experiences and practices related to diagnostic imaging for non-specific LBP in NL, guided by the Theoretical Domains Framework (TDF). Data were collected using in-depth, semi-structured interviews. Transcripts were analyzed deductively (assigning text to one or more domains) and inductively (generating themes at each of the domains) before the results were examined to determine which domains should be targeted to reduce imaging.

**Results:**

Nine family doctors (four males; five females) working in community (n = 4) and academic (n = 5) clinics in both rural (n = 6) and urban (n = 3) settings participated in this study. We found five barriers to reducing imaging for patients with NSLBP: 1) negative consequences, 2) patient demand 3) health system organization, 4) time, and 5) access to resources. These were related to the following domains: 1) beliefs about consequences, 2) beliefs about capabilities, 3) emotion, 4) reinforcement, 5) environmental context and resources, 6) social influences, and 7) behavioural regulation.

**Conclusions:**

Family physicians a) fear that if they do not image they may miss something serious, b) face significant patient demand for imaging, c) are working in a system that encourages unnecessary imaging, d) don’t have enough time to counsel patients about why they don’t need imaging, and e) lack access to appropriate practitioners, community programs, and treatment modalities to prescribe to their patients. These barriers were related to seven TDF domains. Successfully reducing inappropriate imaging requires a comprehensive intervention that addresses these barriers using established behaviour change techniques. These techniques should be matched directly to relevant TDF domains. The results of our study represent the important first step of this process – identifying the contextual barriers and the domains to which they are related.

**Supplementary Information:**

The online version contains supplementary material available at 10.1186/s12875-022-01751-6.

## Background

Low back pain (LBP) is extremely common; it is widely understood to be a leading cause of disability [1] and is among the top five reasons that patients visit their family doctors [2]. As a result, LBP is responsible for substantial economic and social burden [3–6]. While the prognosis for episodes of LBP is generally favorable (most recover within six weeks), some experience pain for up to one year.

International, evidence-based guidelines for the treatment and management of LBP have been established for some time [7]. They recommend that physicians should first assess the patient for evidence of rare cases of specific spinal pathology or radicular syndrome. Only if they suspect the patient might have one of these conditions should they consider imaging as indicated for the specific condition. The remaining cases are considered non-specific low back pain (NSLBP) or low back pain that is not attributable to a recognizable, specific pathology [3]. For these patients, investigations are not required; management should include reassurance, advice to remain active, simple analgesics, and self-care strategies. If patients fail to improve after six weeks, they should be referred to additional conservative care options such as exercise therapy, cognitive behavioural therapy, or chronic pain management programs [7].

Unfortunately, in most cases, patients do not receive care that aligns with these guidelines [8, 9]. This results in poor health outcomes for patients and unnecessary costs and resource use for health systems [10, 11]. One of the main drivers of unnecessary health system costs and resource use in the management of LBP is an overreliance on diagnostic imaging (DI) (e.g., lumbar spine x-ray, CT, or MRI) [12–14]. Roughly 90% of LBP presentations in primary care are NSLBP [3, 15] that should not be imaged as it isn’t useful and introduces potential harm to patients via unnecessary radiation exposure and inappropriate procedures due to incidental findings [12]. Nevertheless, up to half of all requests for lumbar spine imaging are estimated to be inappropriate [16–21]. As a result, one of Choosing Wisely Canada’s key de-implementation campaigns targeting healthcare providers focuses on reducing unnecessary lumbar spine imaging.

Improving uptake of clinical practice guidelines for LBP, thereby reducing unwanted behaviours such as inappropriate imaging requests for NSLBP patients, requires that we understand the full scope of why the behaviour is occurring [22, 23]. This will involve a detailed assessment of the barriers and facilitators to performing the guideline-recommended behaviour so that we can select behaviour change strategies for our intervention that will appropriately address those factors. Ideally, this process should be theoretically-driven using a framework of established psychological theories of behaviour change, [22–25] such as the Theoretical Domains Framework (TDF) which has been used to identify barriers and enablers of behavior change in a variety of contexts [26–29]. Originally developed by Michie et al., [28] the TDF is comprised of a comprehensive assortment of behavior change theories and constructs synthesized into 14 key domains [26–29]. The importance of each domain depends upon the behaviour under study and related contextual factors.

A number of potential barriers to reducing imaging for LBP have been reported in the literature including both practitioner and patient-related factors [30, 31]. A recent systematic review found that for LBP imaging guidelines, reported barriers included pressure from patients requesting an image or wanting a diagnosis, physicians’ beliefs that providing a scan will reassure patients, and a lack of time during a typical patient encounter to converse with patients about diagnosis and why imaging isn’t needed) [31]. While this information is helpful for intervention design, only one of the studies included in the review used a theoretical framework to guide their work. Using a theory-based interview guide may provide a more comprehensive assessment of barriers by ensuring that we do not miss asking important questions related to known barriers to behaviour change. It is possible that without these specific questions, interview data may be limited only to the most common barriers such as time and resources that readily come to mind and may miss capturing important, but perhaps more subtle, barriers. In addition, none of the studies completed to date are tied to the local context, widely considered responsible for study-to-study variation in the outcomes of implementation interventions [32].

As part of a larger study to develop and evaluate a theory-based intervention to reduce inappropriate LBP imaging, we undertook a TDF-guided assessment to identify the barriers and facilitators to following imaging guidelines for NSLBP among family doctors in Newfoundland and Labrador (NL).

## Method

### Design

This was an exploratory, qualitative study describing family doctors’ experiences and practices related to diagnostic imaging for LBP. We used the Atkins et al.[26] guide for applying the TDF to assess barriers and enablers to behaviour change. A description of how we applied the TDF in our data collection and analysis is described below.

### Participants

Eligible participants included family doctors practicing in NL who were treating patients with LBP. Purposive sampling was used to identify study participants. We identified family doctors through an established practice-based network (Atlantic Practice Based Research Network) – a group of clinicians who have agreed to be contacted to participate in primary care research. To help ensure a diverse range of perspectives, we sought participants practicing in both urban and rural environments, as well as both academic and community settings. We planned to recruit and interview 10–15 family doctors or until we reached data saturation – the point at which no novel information was being contributed by additional participants [33].

### Data collection

Following previously established methods outlined in the TDF Guide [26] and other studies using the TDF for barriers assessments, [34] data were collected using in-depth, semi-structured interviews. Potential participants were emailed an invitation to participant in a study investigating family physicians views on imaging for LBP by a local researcher (AH). If interested, they were provided with additional information about the study (including the reasons for the study and our interest in the topic) and an interview time was scheduled.

The interview guide (please see Additional file 1) included 1–4 questions per domain for a total of 31 questions. Prompts were provided in the interview guide to assist the interviewer in clarifying participants’ responses if needed.

Interviews were conducted by two female healthcare researchers (PhD and Master’s – prepared) with experience and training in qualitative interviewing (AH, AP). An undergraduate student (RL) also attended some interviews to take field notes. Otherwise, notes were taken by the interviewers. Interviews were conducted in-person (at the participant’s or interviewer’s place of work) or via telephone, whichever was private and most convenient for the participant. They took approximately 1h to complete, and participants were provided a CAD $100 gift card honorarium (paid using funds from the CIHR grant supporting this research). All interviews were audio-recorded and transcribed verbatim; participants were not provided opportunity to review their transcripts.

### Data analysis

To begin, coders read and reread the transcripts to become familiar with the data; they began coding after we had completed and transcribed three interviews. Using the TDF to generate a framework for content analysis, researchers analyzed the data deductively (assigning text to one or more domains) and inductively (generating themes at each of the domains) [26].

#### Deductive analysis

Under the direction of a TDF expert (AMP), two researchers (AP, RL) were trained to code data from the transcripts into TDF domains. They used transcripts from a previous study (on a different topic but using the same TDF coding scheme) to practice coding. From this work, they created a codebook specific to the current study that served as a guideline and reference to ensure accuracy and consistency.

The codebook contained (1) the coding strategy and (2) a table of coded text which defined, for coders, a clear method for making decisions on whether and how much text to code, which domain was appropriate, and how to deal with any disagreements. Please see Additional file 2 for the codebook developed during the training exercise.

Using the codebook, AP and RL coded all transcripts in NVivo V.12. They coded one pilot interview simultaneously to consensus, with access to an expert coder (AMP) as needed. Using the second pilot interview, they coded independently and calculated Fleiss’ kappa (κ) for each domain to assess reliability of coding. After this, they began coding independently. All interviews were reviewed together and coded to consensus.

#### Inductive analysis

After the data (quotes from interviews) were coded into TDF domains, we generated themes at each domain, phrased as belief statements, that represented important beliefs about a barrier or enabler to the target behaviour common across participants responses (e.g., “I sometimes image NSLBP patients because I don't have the time to explain to them why imaging is unnecessary”). All belief statements and broad themes with supporting quotes were reviewed by the second coder and a TDF expert (AMP). These data were then further analyzed to identify specific barriers/enablers to change.

Finally, the results were examined to determine which of the domains and associated beliefs should be targeted to reduce imaging for LBP. These decisions were made through consensus discussion between the researcher responsible for theme generation (AP) and a TDF expert (AMP); it was subsequently reviewed with the second coder (RL), a key knowledge user (KAB), and the larger research team. Domains were considered relevant if they met any of the following conditions:

- A majority of participants (in our case 5 or more) expressed a belief that contradicted guideline recommendations thereby indicating a lack of understanding or practice of evidence-based guidelines.
- A majority of participants described the same or similar barrier to following imaging guidelines.
- There were a mix of views expressed on a particular issue (for a particular domain) indicating the presence of conflicting beliefs.
- A participant(s) reported a belief that could potentially have a large impact on the target behaviour.
- A participant(s) expressed a belief that they perceived to be a major clinical concern or that they were particularly vocal about (determined by considering the amount of text taken up discussing the issue as well as emphatic or emotional speech).
- Clinical experts on the research team felt strongly that the beliefs expressed at a particular domain represent an important clinical issue.

## Results

### Participants

Nine family doctors (four males; five females) participated in this study. At the time of the interviews these participants were working in community (n = 4) and academic (n = 5) clinics in both rural (n = 6) and urban (n = 3) settings. Seven of nine participants had no previous interactions with the interviewers; two had a previous working relationship with them. None of the participants we contacted refused participation or dropped out. We initially planned to recruit and interview up to 15 family doctors. However, after completing and coding eight interviews we felt that we had reached saturation. To test our assumption, we interviewed one additional participant (adding some additional prompts in an attempt to elicit new information). Since no novel information was added after this interview, we did not pursue additional participants. The team did not feel it necessary to complete any repeat interviews.

### Interrater reliability – Domain coding

Interrater reliability at each domain ranged from a low of κ = 0.67 (SD = 0.32) to a high of κ = 0.92 (SD = 0.21) (with 9 of 14 domains reaching κ = 0. 75 or above) thereby demonstrating substantial to almost perfect agreement [35, 36]. However, while interrater reliability was calculated to help ensure consistency between the coders, all interviews were coded to consensus (100% agreement).

### Relevant domains

Our analysis revealed a number of barriers related to the following domains: 1) beliefs about consequences, 2) beliefs about capabilities, 3) emotion, 4) reinforcement, 5) environmental context and resources, 6) social influences, and 7) behavioural regulation. We generated 49 themes, phrased as belief statements, from the data coded at these domains. Table 1 presents the specific beliefs together with illustrative quotes for each of the relevant domains. Overall, five main barriers to reducing imaging for patients with NSLBP were evident in the data: 1) negative consequences, 2) patient demand 3) health system organization, 4) time, and 5) access to resources.

**Table 1:** Summary of relevant domains (including belief statements and supporting participant quotes)

Domain	Specific belief (frequency)	Sample quotes
Beliefs about consequences	Managing patients with NSLBP with imaging is a waste because it won't help patients or change their course of treatment; most will get better on their own. (6)	What do you think would happen if you managed patients with non-specific low back pain with imaging? Physician: “Nothing. I don’t think there would be any change in outcomes…Fortunately, most people get better anyway.”—LBP001
		Physician: I would create a lot of paper work, and I would create more time problems for myself reading all the x-rays. It wouldn’t help the patient’s pain and it wouldn’t resolve anything.—LBP002
	Ordering images for NSLBP is a waste of time, costly to the health system, and clogs up the waitlist for patients who do require images. (7)	“Negative things would include a crippling cost to the healthcare system”—LBP005
		“I guess negatively, you’d be clogging the system. You know, the resources to x-ray everybody’s back for something that’s, you know, you look at it – what’s the number I needed to treat as such, or the number needed to do, to pick up something. It’d be phenomenal, right? So it’s kind of an irrational thing to do as a policy.”—LBP006
	Imaging exposes patients to radiation which poses a risk to their health. (7)	“Um, and I think that, you know, there are risks associated with doing a whole bunch of x-rays. I think radiation exposure is an issue, um, and so I think we always have to be aware, you know, what is the necessity for doing imaging – especially CTs.”—LBP003
		“I don’t want to expose people to radiation…you don’t want to be imaging…and taking up time for the patients that are just like unnecessary, so, and then like it adds up right? Every time you’re like ‘oh, it’s just one x-ray’, like next thing you know, someone’s had like a hundred x-rays. Like that’s a lot of radiation. Um, so definitely over the long-term, doing like, doing imaging can be an issue. So I think that managing without imaging has that benefit as well from a patient safety point-of-view.”—LBP007
	If I image a patient with NSLBP I may find something serious that I would have otherwise missed or avoid contributing to the development of chronic pain. (3)	I do feel like there is, you know, there is a segment of the population that they are so convinced that this pain is not going to go away…that I will end up doing an x-ray earlier than I would because I feel like if I leave it for six weeks then they will develop chronic pain.”—LBP003
		“You would like to have…every kind of avenue covered. So even if I’m 98 per cent certain that this is non-specific low back pain, it may not be. It could be a small chance that there’s something else starting up that’s going on here, I’m just missing it because I’m not doing an x-ray. So that does nag at you.”—LBP006
	Using imaging to manage NSLBP leads to overdiagnosis due to incidental findings which may cause increased suffering for the patient. (4)	“Incidental findings could show up which require further investigations or tests or interventions, which otherwise not have shown up and actually turn out to be benign.”—LBP005
		“You find other incidental findings that are irrelevant and then you sometimes…sort of lead the patient down the garden path of other investigations.” LBP008
	Ordering images for NSLBP decreases patient anxiety and frustration and increases patient satisfaction. (6)	“Sometimes people get really frustrated and…the negativity and the angst that its creating [makes it] worth doing the x-ray because the negative impacts of what it’s doing to them psychologically may outweigh the balance of, you know, ‘I really don’t think this is going to show anything and I don’t think it’s going to be helpful’.”—LBP004
		“I’m not certain but a possibility is that it would be more reassuring to patients. Despite…that most of them are reassured there’s probably some that are…not completely reassured by what I say and so there a might be a little bit more reassurance [provided by the imaging] that we’re not missing something serious.”—LBP009
	Ordering images for patients with NSLBP saves me time because I can avoid explaining to patients why they don't need one. (3)	“You’d spend less time [on the appointment] because it takes time to talk somebody out of a test than it does to give them the test. Which, could be one of the reasons why people are getting tests, right?”—LBP006
		“It’s a lot faster to fill out the slip and send someone for a test than it is to talk to them and educate them and talk about weight reduction and muscle strengthening and what’s their diet, like very time consuming so it’s so much easier to do tests than to not do tests.”—LBP008
	The radiation risk associated with x-ray is actually very small. (1)	“Realistically we probably over-emphasize the risks of radiation sometimes to our advantage. The measurable risk of the radiation from the x-ray, a plain film x-ray is essentially non-existent. The risk of radiation from a CT scan is much, much greater than x-rays but even the risk, the measurable risk of cancer is pretty small.” LBP009
	If I ordered an image for all cases of NSLBP my colleagues would look down upon me. (1)	“I would be practicing outside evidence-based medicine and therefore rightfully be ashamed with myself as a clinician…my colleagues would look down upon me…if I was known to image every single low back pain that came through my door.”—LBP005
	Ordering an image may help me avoid litigation. (1)	“I think that’s, you know, becomes more of an issue…from a litigations point-of-view. That’s another thing that physicians are, kind of, I think more hesitant about because we have access to all this imaging and then I said ‘listen, it looks okay to me. Let’s give it a couple of weeks. If it gets better.’ And they come back in a couple of weeks and it’s worse, and now I image them and they have a fracture and now they’re saying that they missed work for two weeks and if they imaged earlier, they could’ve done this, and they’re…runs into issues with, you know, complaints and things like that. And I think that those play in the minds of physicians.”—LBP007
Emotion	I feel comfortable managing patients with NSLBP without imaging. (6)	how do you feel about managing a patient with non-specific low back pain without imaging? Would you be worried or concerned? R: “No, not at all.”—LBP004
		“Whenever I decide that I don’t want imaging like I’m, I feel confident in my decision, I don’t kind of think about it after and say ‘oh maybe I should’ve’.”—LBP007
	I worry about not imaging. (3)	“I spend a lot of time worrying that I’m doing the wrong thing and that I’m… missing something that could be serious.—LBP003
		“There’s still an element, in physicians, of a fear of missing something bad, even with reassuring, all reassuring signs and symptoms, you can never say, I say this to patients as well, I can never say 100% in medicine.”—LBP009
	I am fearful of not imaging. (1)	“There’s also this culture of being right a lot and so if you do miss something serious then it’s just frowned upon negatively, there’s the possibility of lawsuits and increase in malpractice over the years, more and more lawsuits all the time and this is our fear of being sued, is a factor as well.”—LBP009
	I find it gratifying to avoid imaging. (1)	How important is it to you to manage patients without imaging? Physician: “It’s a matter of pride. I want to do the right thing so it’s important to me.”—LBP002"
	I would feel ashamed of routinely imaging NSLBP patients. (1)	I would be practicing outside evidence-based medicine and therefore rightfully be ashamed with myself as a clinician. -LBP005
	I am not emotionally affected by managing patients with NSLBP without using imaging. (1)	“I don’t think I have much emotional attachment either way around imaging.”—LBP005
Beliefs about capabilities	I am confident that I can manage my patients with NSLBP without imaging. (6)	“For the most part I find it easy. I feel like if you take time to do a good history and physical and explain to the patient what you feel is going on, advise the follow-up appointments in a few weeks. Know that they can call if it’s getting worse. Like I feel like once you kind of reassure the patient, they’re pretty agreeable to it.”—LBP007
		“It’s something that I put a lot of thought into and I don’t find it hard to do on a day-to-day basis, you know?”—LBP005
	I do not always feel confident managing patients with NSLBP without imaging and may consult a colleague for reassurance. (1)	"If I’m being realistic and I think about how I am in reality, I think the biggest problem is feeling confident in my decision. Often I will… I will refer someone to like an adjunct professional, like a physiotherapist or a chiropractor – just so somebody else is looking at it… to make sure that there’s not something that I’m missing and I’ll often…run it by colleagues…just to feel…more confident.- LBP003
	It is sometimes difficult for me to convince patients that an x-ray is not indicated. (1)	"The difficult part is sometimes dealing with the patient who very much wants imaging and sometimes they’ll look at you and say ‘I’m not leaving this room until I can get an x-ray’. So, that’s when the decision becomes more difficult because sometimes you’re like…’Is this x-ray really worth the fight’?” -LBP007
Reinforcement	Years of experience in managing patients with NSLBP help physicians to avoid imaging. (4)	Are there a lot of cases where you feel you can’t convince them? “Not usually. Sometimes experience helps with that.”—LBP002
		“I think that being a physician in my first five years…I probably image more than I will in 20 years’ time because of a fear that I am going to miss something.”—LBP003
	My previous experiences with either missing a serious problem or picking up on a problem I didn't suspect may have increased my use of imaging. (3)	“I think it was probably after…it might’ve been three months by the time I imaged that one…It turned out to [be] a cancerous mass in the muscle that we thought was muscle strain. So that would push me to want to probably order a little bit sooner than I otherwise might.” LBP005
		“There has been an occasion where I’ve found an aortic aneurysm that wasn’t ruptured but was asymptomatic by taking a plain x-ray. Historically, most of us have picked them up by doing x-rays on backs – for back pain – which wasn’t necessarily aneurysm pain, it was just low back pain. And we’ve seen ‘this person’s got a 5 cm aneurysm on the x-ray’. Because you see calcification come out. So, I’ve picked up aneurysms that way, which were significant and, um, that is…that kind of thing, right.” LBP006
	The way I am paid (more visits = more money) discourages me from taking the time required to follow current guidelines re: imaging for NSLBP. (3)	“In fact…the system would reward me if I just imaged everybody. If somebody came in and said ‘my back hurts’ and I handed them an x-ray slip and, you know, and a prescription for Tylenol 3 s, they’d be out of my office in two minutes. Whereas I take…10 min to do a really good history and…physical exam and…10 or 15 min to explain…why they don’t need [imaging and/or narcotics]…and how to appropriately manage this and what they need to know, and when they need to come back. And so…there are very significant systemic disincentives to practicing good medicine, particularly in this respect, I think.”—LBP003
		“Physicians are not remunerated well in this province so they have to see a lot of people and the faster they do it the better it is for them.”—LBP002
	There are no system-level incentives to encourage physicians to reduce imaging for NSLBP. (4)	“There is no incentive in our organization to be good.”—LBP004
		“Nothing, not even internal to our practice that we look at about comparing imaging ordering rates between colleagues and things so.”—LBP009
	I haven't had any negative experiences (e.g., missing a serious health problem) related to not imaging NSLBP patients. (5)	“I haven’t had the bad experiences in the sense that I haven’t had something that kind of bit me in the butt. Like, whenever I manage without imaging, they tend to improve, most times I don’t even see them by six weeks. They’re telling me that everything is fine. So that’s been kind of reassuring…”—LBP007
		“I can’t think of an example in 16 years of practice and in my residency prior to that where a delay in ordering imaging resulted in a delay in diagnosis of something serious with back pain.” LBP008
	In my experience, imaging has not resulted in benefits for my patients with NSLBP which reassures me that I do not need to image patients. (2)	“In my experience it hasn’t been widely beneficial, so why do it?” LBP005
		”The patients that I have had a suspicion of something more serious have not had anything serious. There’s a patient I had that had urinary retention that I was worried about a cauda equine syndrome but…[they didn’t]. This urinary retention was probably a result of their opioids. It was…a pretty major undertaking to get her a CT scan and so that practice has made me less likely to order more advanced imaging.”—LBP009
Environmental context and resources	I can easily order images for NSLBP; there are no barriers to ordering images in my clinical practice environment. (7)	“Where I work there’s no barriers to ordering imaging.” LBP002
		“I can get a cat scan from my office today or tomorrow. We have really easy access to imaging here.” LBP004
	I sometimes image NSLBP patients because my patients without insurance cannot access appropriate treatment modalities/professionals in my community and don't improve. (4)	What might make it difficult to manage a patient with non-specific low back pain without imaging? “Here is the challenge and I run into this very frequently. You can try to refer to therapy, and then you will want to do your physio, chiro, and so forth. The problem is if [the patient has] no money or [they] do not have any insurance. If you got an insurance plan, you have money, I can have you into the chiropractor the next day, or two days, and physio is the same way. But, if you don’t, that’s my challenge.”—LBP001
		“One of the problems we face here is that we don’t have ready access to physiotherapy for outpatients for people who are not on private insurance. So I have a patient with sciatica who is now six months waiting for physio. And I’ve had to image her because we’re six months into this…- LBP006
	I sometimes image NSLBP patients because I don't have the time to explain to them why imaging is unnecessary. (5)	“The way medicine is practiced is not necessarily conducive to thinking through things. Management is often hurried and…they’re happy to get the x-ray…and you get out quick—LBP006
		are there any other competing tasks or time constraints that might influence whether or not you use imaging for a non-specific low back pain patient? “The next patient. How far behind I am in the clinic, how much time I can take to reassure a patient,…So, I think, so is the time available would be the main thing.”—LBP009
	The way I am paid makes it hard for me to take the time needed with patients in order to avoid imaging. (2)	“Physicians are not remunerated well in this province so they have to see a lot of people and the faster they do it the better it is for them.”—LBP002
		“I think that if I practiced medicine the way that the system wants me to, everybody would get imaging and opioids.” Because of time? “Absolutely. Time.”—LBP003
	I sometimes image patients with LBP because it is so easily accessible to me and my patients. (1)	“Sometimes the convenience factor makes it, you know…easier…Sometimes convenience makes people slacker…” LBP004
	The high cost of imaging to the health system makes me think more carefully about whether an image is required. (1)	“The financial that the state that the province is in currently [is] a minor factor in my decision making.” LPB009
	I don't think ease of ordering influences my decision to order. (1)	“I doubt we’re really influenced much at all by ease of ordering, so ease of completing the paperwork.” LBP009
Social influences	Patient (and family member) requests for images influence my image-ordering decisions. (7)	“Sometimes I’ll do it because it is easy and because the patient wants it and I just run out of energy to argue.” LBP003
		“The difficult part is sometimes dealing with the patient who very much wants imaging and sometimes they’ll look at you and say ‘I’m not leaving this room until I can get an x-ray.” LBP007
	My colleagues do not influence my image-ordering decisions. (5)	Do you ever discuss the case with colleagues before deciding whether to manage a patient without imaging? “Nope.” LPB001
		Is there ever a time where you might discuss with your colleagues before your deciding whether to manage one of these patients without imaging? “No I wouldn’t really discuss that with colleagues.” LBP008
	Patients and/or their family members pressure physicians for imaging. (7)	“Except when the patient is persistent and you have to use all your might in order to persuade them not to have imaging.” Interviewer: Would that be a common occurrence do you think? “Yes. ‘I’m just here for an x-ray doc.’ ‘I’ve just come to get an x-ray on my hip I got back pain.’ Patients come with that expectation.” LBP002
		“Usually the patient wants an x-ray….’cause everyone wants imaging. So then I just have a fairly lengthy discussion…” LBP007
	I sometimes order imaging for NSLBP when referring my patient to other HCPs, at the request of other providers, or when my patient requires that documentation for an insurance company or workers compensation. (4)	“Sometimes when there is insurance or when workers compensation is involved that imaging might be appropriate.” LBP002
		“I think sometimes I will order it if I’m going to be referring ‘cause I think that somebody else might be looking for it. I don’t hardly ever refer to surgeons, but I will refer to neurology. But also sometimes, um, you know, if I’m referring to physio or a chiropractor to just say ‘ok, well we did do the x-ray and the x-ray didn’t show anything’. LBP003
	My image-ordering practices re: NSLBP are sometimes influenced by discussions with my colleagues and/or their ordering practices. (6)	“My colleagues would look down upon me, and rightly so, I think, if I was known to image every single low back pain that came through my door.”—LBP005
		“There’s a discussion, or a talk a lot, especially in the academic circles I guess of how, in order to see patients quickly a lot of the faster docs will just order far too many imaging tests and it’s just seen as less, it’s not as good medicine and so ordering fewer tests is seen as higher quality medicine in those circles so.”—LBP009
	Patient pressures for imaging don't impact my ordering behavior. (2)	“Even if they’re really insistent, you would still say ‘look, you know, if things are not getting better with time, yes we would do that but right now there’s nothing alarming’.” LBP004
		“I don’t really feel like it takes me a ton of time to convince patients that they don’t need an imaging test.”—LBP009
	Patients sometimes influence me to delay imaging. (1)	“Then there’s some other people that it’s at the 6 week mark and you’re like okay you’re back and you’re not better or so maybe you know um maybe we’ll order an x-ray and the patient goes do I have to doctor I don’t like to get x-rays can I just carry on with physio for another few weeks and do more stretches or I’m trying to get back into walking or whatever…”LBP008
Behavioral regulation	We need easy access to good patient education materials to provide to our patients. (8)	“Maybe a one-page little hand out. ‘Why didn’t I get an x-ray today?’ something like that. A little resource sheet and attached to it back care exercise protocol.” Written information for the patient makes it easier."—LBP002
		“There would be you know…somewhere that the person can go to, that would link in about eating healthy and types of exercise you could even do in one’s home…it is just simpler for patients to go to one place that they could read about back health…I think most people are going online for stuff these days…so if there’s good websites and stuff that they don’t have to go on doctor google that would be much better."—LBP008
	Access to the healthcare professionals and community-based resources for the treatment of LBP would help physicians to avoid imaging. (8)	“Having a physiotherapy-based chronic disease management program I think would be really good for backs."—LBP004
		“I think to have more accessibility to the allied health care services sometimes because a lot of times patients feel like you’re not doing anything."—LBP007
	Improved clinical tools would help physicians avoid imaging for LBP. (3)	“A standard protocol. Like this is how we manage non-specific low back pain. So that everyone’s on the same page. I think that would be super important"—LBP007
		“I guess the problem with a lot of online resources is that people don’t wanna go to a hundred different places and family doctors can’t keep track of a million type of resources. So, the more things are integrated into primary care systems base and stuff the easier it is for people to find."—LBP008
	Improved access to other types of diagnostic imaging would help physicians reduce x-ray and CT imaging rates. (2)	"I’d order fewer CTs if I had access to MRIs and more appropriate imaging"—LBP005
	Using fear-based strategies to curb patient demand will help physicians reduce imaging for LBP. (2)	"Realistically we probably over-emphasize the risks of radiation sometimes to our advantage when we don’t want to order. Certainly in x-ray I mean the risk of, the measurable risk of the radiation from the x-ray, a plain film x-ray is essentially non-existent. But, we mention that and with CT it’s, even the CT risk is pretty small when you quantify it. We do discuss that though when we’re talking about the risks of imaging. We do seem to get some mileage out of it I guess."—LBP009
	Information about their ordering behaviors may help physicians avoid over-imaging for LBP. (2)	"I think it’s more just look at your current practice and see how that fits with Choosing Wisely, how it fits with current guidelines because if you do think like ‘ok, maybe I’m not doing this right’, then you do need to go back and look at what current guidelines are there to guide me. You know, what is Choosing Wisely saying about this? And you know, modify your practice based on that. Because lots of times, I mean, if you’re in practice twenty years you do things that you’ve always done and maybe there are new things, maybe there are ways to change"—LBP004
	I encourage patients to do the work required to take care of their backs in order to help reduce need for imaging. (1)	"I put the onus on the patient to get better. If they are just sitting in an armchair watching TV and not being active they can’t expect to get better."—LBP002
	Increased compensation for physicians will encourage them to take the time required to avoid imaging. (1)	“The truth of the matter is that I literally cannot afford to practice good medicine in rural Newfoundland. I literally cannot afford it.” I: And is that because the education that you want to be providing takes time? “Yeah."—LBP003
	I don't use any strategies to help me reduce imaging. (1)	Do you have any steps or strategies that would encourage you to manage patients without imaging? R:“No, I mean it’s not a first-line part of what we do for back pain”—LBP004

#### Negative consequences

Physicians reported imaging cases of NSLBP because they feared missing a serious illness (beliefs about consequences, emotion). Related to this was a fear of litigation that could result if a serious condition was missed (emotion). Over a third of our sample reported that previous negative experiences (e.g., missing the presence of an underlying serious pathology in a previous case) play a role in their decision-making (reinforcement). Some believed they can use imaging as a sort of “fail-safe” to pick up serious conditions they might have otherwise missed (beliefs about consequences).

#### Patient demand

Seven of nine participants reported that their patients and/or family members pressure them for imaging and that patient pressures of this nature influence their image-ordering decisions (social influences). They also felt that, in some cases, ordering the image will be beneficial for patients by reducing anxiety and frustration and increasing patient satisfaction (beliefs about consequences). Related to this, some physicians reported difficulty convincing patients that they don’t need an image (beliefs about capabilities). Patient demand can also become a factor when physicians within a clinic do not adhere to the same imaging practices. Physicians reported that patients sometimes pressure them for images because other doctors at their place of work image more liberally and patients believe that to be a higher standard of care (social influences).

#### Health system organization

Many participants reported a lack of system-level rewards for not imaging patients with NSLBP (reinforcement). Further, some felt the health system punishes physicians for using conservative ordering practices (reinforcement). They explained that because of the way physicians are remunerated in this province (fee-for-service model), they lose income when they take the time required to explain to patients why imaging isn’t necessary and counsel them on alterative therapies for treatment. Because of this, it makes it difficult for them to take the time necessary to avoid imaging (environmental context and resources). Related to this issue, some physicians reported they sometimes image patients with NSLBP when referring a patient to other healthcare providers (because they think it will be required), at the request of other providers, or when their patient requires that documentation for an insurance company or workers compensation.

#### Time

Participants reported that it takes much longer to explain why an image is not needed than to simply order an image (beliefs about consequences). They don’t feel they have adequate time to convince patients that they don’t require imaging in the run of a busy clinic day (environmental context and resources).

#### Access to resources

Physicians reported they sometimes image because their patients do not have the means and/or opportunity to assess appropriate treatment modalities and/or health professionals and, as a result, their condition fails to improve (environmental context and resources). They explained that access to publicly-funded physiotherapists is very limited and that wait times to see these practitioners are often prohibitively long. Compounding this issue, is the fact that their patients often don’t have insurance plans that would cover some or all of the costs associated with private practitioners (e.g., physiotherapists, massage therapists, and chiropractors) and therefore can’t afford these treatment venues. Finally, in some rural environments, there is not always a private practitioner available. Family physicians believe that alternative/allied health professionals and community-based programs for the treatment of NSLBP including the addition of other health professionals to the clinic environment or a physiotherapist-based low back pain management program would encourage them to avoid imaging their patients with NSLBP (behavioural regulation). A large majority of our participants also felt that improved access to quality patient education materials would encourage family physicians to image more conservatively. They suggested a one-page handout to give patients during an encounter or a trusted, online repository for evidence-based patient education materials (behavioural regulation). A smaller number also wished for improved clinical tools (behavioural regulation) such as an evidence-based algorithm that could be inserted into existing electronic medical records.

### Irrelevant domains

The remaining seven TDF domains were not considered relevant and included: 8) knowledge, 9) skills, 10) social professional role and identity, 11) optimism, 12) intentions, 13) goals, and 14) memory, attention and decision-making. The data coded at each of these domains revealed 34 specific beliefs, presented in Table 2 with illustrative quotes. Below we provide a brief summary of these beliefs which did not indicate the presence of any barriers to reducing imaging for patients with LBP.

**Table 2:** Summary of irrelevant domains (including belief statements and supporting participant quotes)

Domain	Specific belief (frequency)	Sample quotes
Knowledge	The guidelines indicate that I should not image patients with LBP unless their pain has lasted for over 6 weeks, and/or I suspect red flags, and/or they are not candidates for surgery. (8)	“to the best of my knowledge, the guidelines are: if there’s no red flags and the pain doesn’t resolve within six weeks, then plain film might be warranted. And I think certainly if a patient then develops any more severe neurological symptoms or something like that, then potentially move on to a CT.”—LBP003
		“unless there’s red flags you probably wouldn’t consider any imaging for about at least 6 weeks”—LBP008
	Recommendations suggest I should not routinely image LBP patients. (1)	“Choosing Wisely…is is very much against the doing routine imaging…” LBP007
	I should provide conservative care to NSLBP patients. (4)	“you try to educate them on the hygiene of that care. Then, in terms of medication, keep it simple. I’m sort of like a Tylenol type person, sometimes I will use anti-inflammatory, and again, this is keeping with the risk of the person. I will keep it at the lowest dose with the minimum time possible…”LBP001
		“many will recover with the conservative therapies I’ve outlined and in the absence of red flags, that is appropriate.” LBP005
	I am aware that there are clinical practice guidelines for the treatment of NSLBP. I believe that guidelines and recommendations for the management of LBP are evidence-based. (7)	“The one that I have that I tend to use is the one that the College of Physicians and Surgeons put out. I think the CPSNL has guidelines for low back pain.” -LBP002
		“I know there are some. I can’t say I’ve totally used them.”—LBP004
	I believe that guidelines and recommendations for the management of LBP are evidence-based. (6)	“Do you believe those guidelines to be evidence based? Physician: Yes.” LBP002
		“Do you believe that these guidelines are evidence-based? R: The ones that I’ve seen are.”—LBP006
	I am aware of the Choosing Wisely recommendations re: reducing imaging for LBP. (5)	“guidelines around lumbar…x-rays…they’re kind of part of the choosing wisely…unless you have any alarming symptoms you don’t need imaging on your back, and you know”- LBP004
		“I’m aware of the imaging guidance around, in the Choosing Wisely recommendations around back pain”—LBP009
	I am not aware of clinical practice guidelines for the management of NSLBP. (2)	“Are you aware of any guidelines about managing patients with non-specific low back pain? R: [pause] I read articles on it. I don’t know if any of them were guidelines.”—LBP005
		“Are you aware of any guidelines about managing patients with non-specific low back pain? Physician: Um, other than Choosing Wisely probably not”—LBP008
	I have no knowledge of or question the strength of the evidence supporting LBP guidelines. (3)	“I suspect that there’s evidence that goes in to producing the guidelines but I don’t think they’ve been studied in a randomized trial setting to determine whether they’re safe.”—LBP009
		“would you know anything about the interpretation of the evidence that supports them? R: No, I don’t”—LBP003
Skills	I believe that family physicians have the training they require to manage NSLBP when they complete their formal education. (6)	“I think we have enough training when we graduate that we should competently, comfortably, and confidentiality manage low back issues.”-LBP001
		“what training would someone need to feel competent in managing patients with non-specific low back pain [without imaging]? R: A family medicine degree.”-LBP005
	I believe that family physicians require good clinical skills (e.g., history-taking, physical exam, symptom recognition) to manage NSLBP patients without imaging. (7)	“I mean, certainly that sort of initial history and physical exam.” -LBP003
		“Stuff that you’d learn in clerkship, right? Which would be basic history, physical.”—LBP006
	I believe that family physicians require good communication skills to manage NSLBP patients without imaging. (3)	“A patient-centered approach, where you can listen to the patient, find out what their experience of the illness is and what their goals are. Um, and also you need to knowledgeable and able to describe their situation from your perspective as a physician as to what you think might be wrong with them, what are the reasons for recommending whatever treatment plan or imaging or other investigations that you might need so that they can make an informed decision themselves. And working with the patient to come up with the best plan that makes sense for them.”—LBP005
		“there’s no question that’s important actually the way we communicate these things to patients probably affects their confidence in us as diagnosticians and managers of their pain as well as their perception of the severity of their problem and their reassurance about the severity of the problem and therefore there, the degree to which they push for imaging, or some other diagnostic test.”—LBP009
Social professional role	Managing NSLBP patients without imaging is a part of doing my job. (9)	“If you are monitoring a patient with non-specific low back pain and you don’t order a CT or an x-ray, do you think you are doing your job? R: Yes! That is my job!” -LBP003
		“If you were monitoring a patient with non-specific back pain and you don’t order a CT or an x-ray, do you think you’re doing your job? R: Oh, absolutely.” LBP007
	Part of my role as a primary care physician involves considering resource utilization and avoiding low-value care. (3)	I think…we shouldn’t be doing any tests we don’t need that are not going to have any value.” -LBP004
		“One of the roles of primary care in the health system is to improve the efficiency of the health system and we do that by triaging appropriately and using our clinical skills rather than relying on diagnostic tests.” LBP009
	As a physician and among my colleagues, using my clinical skills and avoiding unnecessary images for patients is practicing good (evidence -based) medicine. (7)	“Good medicine is educating people and you know, and being aware of guidelines and following them unless you have a reason not to.”—LBP003
		“You wanna be a good manager in the system and you don’t wanna subject patients to unnecessary investigations if they don’t need them.” LBP008
	My involvement with Choosing Wisely has likely influenced by my rate of ordering. (2)	“Well I’m on the Choosing Wisely committee! I’d be pretty sheepish going in, if I’ve got the highest rate of back x-rays. Oh yeah, I mean, part of it is that I’ve, you know, I’m involved with this stuff. That’s helped, you know.”—LBP006
		“Certainly…all the talk around Choosing Wisely recently in the news and my involvement with it…has probably influenced the rate of ordering. It’s not really been formal training but just what we’ve come across in our practices and the news that’s circulating about Choosing Wisely that’s probably influenced yep.” LBP009
	Because I am a salaried provider, I can schedule more time to see patients that FFS docs which may help me to keep imaging rates lower. (1)	“Probably one of the biggest factors I would say that makes my ordering, I suspect less than most other or many other doctors anyways is that I have, I’m a salary provider, I usually schedule a bit more time to see patients than …most fee for service docs would, take a bit more time to examine them and ask more detailed history and counsel them and, so I suspect that likely is the biggest thing that translates into lower ordering than the fee for service docs.” LBP009
	Since I am an older physician, patients may be more willing to follow my suggested treatment. (2)	“Sometimes experience helps with that and bit older physicians, so they tend to believe me.” LBP002
		"Maybe just the fact that I’ve got a bit of grey hair and I speak with more confidence than I used to 15 years ago when I first graduated, it’s more reassuring to patients that what I’m saying is probably true, that might be part of it as well.”—LBP009
Optimism	I think that managing patients without imaging is a good idea. (6)	“I think it’s good. I mean, we shouldn’t be doing any tests we don’t need that are not going to have any value. And I think it’s equally wrong to subject patients to tests that don’t have a real clinical indication.”—LBP004
		“I think it’s a good idea. I think that we over-image to an extreme. It’s bad for the patient, it’s bad for the healthcare system so I think that managing patients with non-specific low back pain without imaging is good overall both for the patient as well as the healthcare system.”—LBP007
Intentions	I intend to manage patients with NSLBP without using imaging. (7)	“As long as they truly meet all those non-specific criteria then, yeah that would be the intent to manage them that way [without imaging].-LBP009
		“ Yeah, so going into it, I plan to not have any imaging. Like unless, like I said, unless there is something specific that I need it.” LBP007
Goals	I want to manage patients with NSLBP without using imaging. (7)	“How important is it to you to manage patients without imaging? That group of patients Physician: It’s a matter of pride. I want to do the right thing so it’s important to me.”—LBP002
		“I think it’s important. Especially the acute cases, like, I feel like as a society we’re dumping way too much towards diagnostic imaging and physicians are forgetting that we worked with our hands long before we had x-rays.”—LBP007
	I want to use resources wisely. (5)	“Are there any personal incentives for you to manage patients without imaging? R: trying not to waste people’s time. Government money.”—LBP004
		“I guess we always just use their slogan like choose wisely, you wanna be a good manager in the system and you don’t wanna subject patients to unnecessary investigations if they don’t need them.”—LBP008
	My priority is to ensure my patients receive the care that they need which may or may not include avoiding imaging. (2)	“Using resources appropriately is an important part of an office visit, you know, I…but there’s a lot of other things you’re trying to accomplish. Some of which is the doctor-patient relationship – the therapeutic relationship – and keeping somebody kind of offside and in cooperating and so sometimes I will sacrifice that for a bigger cause. The bigger cause may be this relationship is going to go south if I don’t…the patient really wants this and they’re anxious and they’re upset. If they don’t get an image they’re gonna be…so to me, that’s a higher order thing to address than the x-ray. So it’s all about, you know, you have to weigh – you’re weighing things out all the time. What’s the priority here? What’s the problem here? Can I not do it without sacrificing something greater? If that’s the case then, you know, I’m trying not to do it but it’s not the most important thing.”—LBP006
		“It’s not really a priority to manage it without imaging, it’s um, I mean priority is the patient feeling better.” -LBP008
Memory, attention, and decision processes	I consider patient characteristics (e.g., age, comorbid conditions), patient symptoms, and/or physical exam findings when deciding whether to order an image. (7)	What aspects of the family practice environment influence your choice of ordering for non-specific low back pain? Physician: “Symptom and physical examination driven. I should be influenced by the symptom presentation, the physical examination, and the results.”—LBP001
		“The patient’s past history,…whether or not they had a malignancy before or what their other comorbidities would be,…there’s also factors like um patient expectations, um like what you’re thinking of in the differential diagnosis.”—LBP008
	I determine if that patient is a surgical candidate to help me decide if I should order imaging. (2)	“My jump out point as I said in the beginning is…[to]] put them in surgical or non-surgical…”—LBP001
		“When I’m ordering a CT it’s probably because…things are not settling out. They’ve got what I think is mechanical low back pain – and I say that simply because that is the vast majority of back pain that comes in. Um, and it’s gone on long enough and it’s been severe enough that it is possible they may be a surgical candidate.”.-LBP005
	I assess the patient for red flag conditions before ordering imaging. (4)	What guides your decision to use imaging with non-specific low back pain patients? R: “Red flags. Length of time they’ve had it.” -LBP005
		“The big thing that goes through my mind is…red flags for back pain, right? So if I’m seeing any… red flags, then I’m going to image”.-LBP007
	I do not automatically order images for patients with NSLBP. (7)	Is ordering a CT or an x-ray an automatic decision or is it something you take time to think about with your patient? Physician: “No. I think about it all the time.” LBP002
		When you do order a CT or an x-ray, is it an automatic decision or is it something you take the time to think about with non-specific low back pain R: “I take the time to think about. It’s not automatic.”—LBP005
	It is not difficult for me to decide whether to order images for patients with NSLBP. (7)	“Generally it’s pretty clear if something’s got to be imaged.—LBP006
		“Most times I would say it’s easy.—LBP004
	I consider the patient's response to previous treatments when deciding whether to order images. (2)	“I’ve seen a patient with sciatica and the first three months, I think I managed her without any imaging. But three months she was still having significant leg pain and I couldn’t really control the pain. So then you start talking about things like imaging.” -LBP006
		“If the anti-inflammatories work – then we’re done. If physio works, then we’re done. If they’re not making significant improvement after a month or two and having tried those things then…and potentially depending on the nature of the pain and its location, and the physical exam – potentially massage or acupuncture, other things as well – then after a month or two I might get an x-ray.” LB005
	I consider resource utilization when deciding whether to order images. (3)	“Its not the *most* important thing but using resources appropriately is an important part of an office visit.” -LBP006
		“The financial that the state that the province is in currently makes us think carefully about the image and tests we order. It probably shouldn’t, but it’s a minor factor in my decision making.”—LBP009
	Ordering imaging is a difficult decision to make. (1)	Is it typically an easy or a difficult decision to make? “I find it really hard, yeah. I find it really hard.”—LBP003
	I try to determine if the patients’ pain will persist when deciding whether or not to order an image. (1)	“But then the other huge factor is ‘do I think that this person’s pain is going to persist if they don’t get an x-ray because they believe that it will’?”—LBP003
	If I have any doubt or concern about my patient at all I will image. (1)	“I think sometimes if I am referring and there is a, you know, question in my mind or there’s something, you know, that maybe is not what everybody has but still, you know, wouldn’t fall under the category of a red flag or something like that, then I will sometimes do imaging…”—LBP003
	Having easy access to imaging does not influence my decision making. (1)	“Accessibility is not part of [my decision]. I have the CT available to me 15 min down the road [but] that makes no difference to me.” LBP001
	I use guidelines in combination with my knowledge of the patient's needs to help me decide whether to image. (1)	"You know,… there’s a lot of other things you’re trying to accomplish. Some of which is the doctor-patient relationship – the therapeutic relationship – and keeping somebody kind of offside and cooperating and so sometimes I will sacrifice that for a bigger cause. The bigger cause may be this relationship is going to go south if I don’t…the patient really wants this and they’re anxious and they’re upset. There’s no sense, you know, keeping someone from an x-ray and losing a patient in the sense of losing their trust or their…the two don’t equate.”-LBP006"
	I am more judicious when ordering CT scans than when ordering x-rays. (1)	“I don’t want to give the impression that I just throw around x-rays. But I am even more judicious in my use of CT scans because of the increased cost to the healthcare system because of the increased amount of radiation that the patient will receive in the process.” LBP005

All participants were aware of the behaviours specified by the guidelines for the appropriate management of NSLBP and most understood them to be evidence-based (knowledge). However, a few were not sure of the quality of the evidence supporting the guidelines. Likewise, most of our participants believed that family physicians require good clinical skills (e.g., history-taking, symptom recognition) – acquired over the course of their educational experiences – to manage low back pain without the use of imaging (skills). There was no evidence of disagreement among our participants and no conflicting or incorrect beliefs noted.

For the most part, the family doctors in our sample don’t feel that their ordering decisions are automatic but they also don’t struggle with those decisions. Most reported considering the patient’s history and physical exam findings when deciding whether or not an image is warranted. Other considerations include assessments for red flag conditions and surgical candidacy as well as response to previous treatments and resource stewardship (memory, attention, and decision-making).

All participants felt that managing patients with NSLBP without the use of imaging was a part of doing their job and meant they were practicing “good medicine” (social professional role and identity). Further, most reported that stewardship of health care resources was a part of their role as responsible practitioners and that avoiding low value care (such as imaging the low back) means they are practicing evidence-based medicine (social professional role and identity, goals). There was no evidence of discord or other salient information that leads us to suspect the existence of barriers related to this domain.

The majority of participants felt that, generally speaking, managing patients with NSLBP without imaging is a good idea (optimism). Further, most also reported wanting to manage their patients with NSLBP without imaging (goals) and their intention to manage their patients with NSLBP without imaging (intentions). There were no conflicts in these messages, however, two participants stressed that their priority is to ensure their patients’ well-being and not resource stewardship (goals).

## Discussion

This study used the TDF to conduct a theory-informed, comprehensive investigation of the barriers and enablers to reducing unnecessary imaging for LBP in the Canadian province of NL. Our investigation revealed a number of barriers related to the following domains: beliefs about consequences, beliefs about capabilities, emotion, reinforcement, environmental context and resources, social influences, and behavioural regulation. Overall, five main barriers were evident in the data. Briefly, they are 1) negative consequences – family physicians fear that if they do not image they may miss something serious, 2) patient demand – physicians face significant patient demand for imaging, 3) health system organization – family physicians are working in a system they feel encourages unnecessary imaging, 4) time – family physicians don’t have enough time during a typical busy clinic data to counsel patients about why they don’t need imaging, and 5) access to resources – family physicians reported a lack access to appropriate practitioners, community programs, quality education materials, and treatment modalities to prescribe to their patients. We found that the remaining seven domains in the TDF (knowledge; skills; memory, attention, and decision-making; social professional role and identity; optimism; intentions; and goals) were not relevant to reducing unnecessary imaging.

### Previous research on the determinants of unnecessary imaging

Our findings are largely supported by previous studies that have investigated barriers and enablers to following guideline-recommended treatment and management of LBP in primary care. Two systematic reviews have assessed the literature focused specifically on physician-reported barriers to guideline-recommended imaging. In 2016, Slade et al., [30] performed a systematic review and meta-synthesis of qualitative studies investigating primary care physicians’ perspectives on clinical practice guidelines for LBP including barriers and enablers to their adherence. Building on this work, Hall et al., [31] completed a systematic review that included much of the same literature (plus two additional studies) and used a theoretical framework (the TDF) to guide the analysis. Several of our findings are in line with the results of these previous reviews. Briefly, social influence in the form of patient demand was an important factor in the decision to image for NSLBP, [30, 31] physicians felt imaging would ease patient anxiety and increase their satisfaction with care, [31] time is a barrier to reducing unnecessary imaging, [30, 31] and physicians imaged patients with NSLBP in part because they felt that it could act as a sort of “fail-safe” to protect them against missing a serious underlying condition [30].

There were, however, some important differences between the current investigation and existing literature. For example, unlike the Slade review [30], the participants in our investigation did not lack knowledge of guideline content and did not take issue with the credibility of clinical practice guidelines for LBP. Overall, they were also confident in their clinical skills to manage patients with NSLBP without the use of imaging. That being said, similar to findings noted in Hall’s review [31], the physicians we interviewed all reported some degree of struggle related to avoiding imaging for LBP. In many cases, they believe it is easier to order an image than negotiate with patients in order to avoid imaging. All physicians reported that they either a) don’t have the time to explain to patients that an image isn’t needed and/or b) struggle to convince patients who insist on imaging and/or c) believe that because some patients are reassured by imaging, it would damage the therapeutic relationship to deny it. Interestingly, a review including patient perspectives on imaging for low back pain found that some patients are, in fact, frustrated by imaging – particularly when the results are are inconclusive, don’t provide a clear reason for their back pain, or indicate degenerative or other issues perceived to be permanent or irreversible [37].

Our investigation also found an important barrier related to physician remuneration that has not been noted elsewhere in the literature on barriers to reducing imaging for LBP among family physicians. While physicians in NL are not remunerated for generating a referral, those we interviewed reported that the way they are remunerated (under a fee-for-service model) encourages imaging patients with NSLBP. They explained that under the current fee-for-service model in this province, they lose income by taking the time to explain why imaging is not necessary and counsel patients on alternative therapies for treatment. This finding underscores the importance of completing a context-specific barriers assessment before intervening to change behaviour.

### Implications for research and practice

A number of studies have now assessed barriers to following guidelines in the treatment and management of low back pain in a variety of settings in 10 countries and have identified similar barriers in these different contexts [31]. Our study revealed five important barriers to reducing unnecessary imaging, many of which align with the key barriers noted in previous reviews [30, 31]. Given the convergence of research in this area, it is unlikely that repeating barriers assessments in similar contexts will change these overall findings. Thus, any future research to examine barriers related to imaging guidelines for low back pain should consider focusing on areas that may add new and valuable information to the established knowledge base. For example, researchers may want to consider targeting particular contexts that have been under-studied in current literature such as resource-poor settings, or physicians with higher-than-average rates of imaging. Similarly, when an intervention to reduce imaging is being planned within a local context for which a full barriers assessment has not been conducted, we recommend using the existing literature as a reliable foundation to start selecting potential strategies and consider conducting 3–5 interviews with key contacts to confirm if there are any other issues that might be specific to the local context. These assessments would be undertaken not to generate new scientific knowledge but to optimize an intervention for a particular context.

Several interventions have been implemented to address over-imaging for low back pain [38–42]. However, most have focused on providing information to clinicians to increase their knowledge of the guidelines and have been largely unsuccessful in changing clinician ordering practices [42]. While lack of knowledge has been identified as a barrier to evidence-based ordering in some contexts, [30] the bulk of the evidence in this area shows us that addressing only knowledge via strategies like practitioner education or passive dissemination of guidelines is not enough [38–42]. Rather, moving evidence into practice for this behaviour will require more comprehensive interventions that address the most relevant barriers using behaviour change techniques matched to as many of the implicated domains as possible. Following guidance from the Medical Research Council (MRC), theory-based interventions should be tested in randomized controlled trials (RCTs) that include a robust process evaluation [43]. A randomized design is important to consider wherever possible since we don’t have a good understanding of other confounders related to behaviour change, making it difficult to adjust for them in non-randomized designs. Process evaluations allow us to explain how complex interventions work by examining the processes through which an intervention generates outcomes. When testing behaviour change interventions (BCIs), a process evaluation should be carried out to assess (i) fidelity and quality of implementation of the intervention, (ii) clarify causal mechanisms and (iii) identify contextual factors associated with variation in outcomes [43, 44]. These evaluations are vital for understanding how interventions function in different settings.

Next steps will involve using what is known about the barriers to reducing imaging to develop a comprehensive intervention to improve uptake of imaging guidelines for the treatment and management of NSLBP using the resources developed by Michie et al. to complement the TDF [27, 45, 46]. These include the Behaviour Change Wheel—a systematic guide for designing BCIs and the Behaviour Change Technique Taxonomy – an extensive list of 93 techniques, divided into 16 categories that will form the active components of an intervention [27, 45, 46]. Using this method, an intervention to reduce imaging for low back pain should include behavior change techniques that target domains directly related to specific barriers. Each barrier may be related to a number of different TDF domains and each domain is, in turn, associated with several BCTs. The theory and techniques tool (https://theoryandtechniquetool.humanbehaviourchange.org) has been developed to help researchers map behavior change techniques to identified TDF domains [47–49]. The tool is essentially a heat map in which each cell represents a link between a BCT and a TDF domain with colour-coding used to provide indication of the strength of the evidence link between them. The strength of the links was determined through a literature synthesis study that extracted data from 277 behavior change intervention articles, [47] an expert consensus study including 105 international behavior change experts, [48] and a triangulation study (statistically assessing concordance between the first two studies supplemented by a consensus exercise to reconcile discrepancies) [49]. Tool developers have also established a repository where new behavior change intervention study data can be uploaded and synthesized in order to help keep this tool up-to-date. As such, it is susceptible to change as the evidence base grows. Using the theory and techniques tool, we have included an example of how BCTs could be used to build an intervention targeting barriers to reducing imaging for LBP in Table 3. For the purposes of this example, we have focused on only one barrier.

**Table 3:** Example of how behavior change techniques can be used to build an intervention targeting TDF domains related to barriers to reducing imaging for LBP

Barrier	Domains	Behavior Change Techniques*/Intervention Components
Patient demand	Beliefs about consequences (Imaging reassures patients)	5.3 Information about social and environmental consequences Inform clinicians that patients are frustrated about imaging findings that “don’t show anything”
	Beliefs about capabilities (It is difficult to convince patients that imaging isn’t useful)	8.1 Behavioral practice Use role-play exercise in training physicians to use a new tool to facilitate conversation about why images aren’t useful
	Social influences (patients pressure me for images)	3.1. Social support Peer counselling or consults established to help work through issues with insistent patients

We have chosen BCTs from among those with “strong” evidence links to each TDF domain in the table

We will complement this process by also involving family physicians and other relevant stakeholders who can advise the research team on the acceptability and plausibility of successfully implementing the intervention. This intervention will be tested in a RCT that includes both a rigorous process evaluation (to identify causal mechanisms) and fidelity assessment (to determine the extent to which the intervention was implemented as intended).

### Strengths

As recommended by a host of national health and research organizations (e.g., the National Institute for Healthcare Excellence, the MRC, Health Canada, Canadian Institutes of Health Research, and the Quality Enhancement Research Initiative), [43, 50–53] this study used a theory-informed approach to investigate the barriers and enablers of unnecessary imaging for NSLBP. While both physician and patient-related barriers to reducing imaging for NSLBP have been noted elsewhere in the literature, [30, 31] only one study that we are aware of used a theoretical framework to guide their assessment [54]. Our study was designed using the Atkins et al.guide [26] on how to apply the TDF to the development of data collection and analysis methods for assessing barriers and enablers to behaviour change. Using this approach allowed us to produce results that can be used to guide the development of an intervention that will appropriately tackle barriers related to each of the TDF domains.

Additionally, we used a rigorous approach to data analysis as recommended by Atkins et al. [26] in their guide to applying the TDF to barriers assessments which included double coding the transcripts, training coders extensively, oversight of analysis by a professional expert in the TDF, review of the results by a family physician team member in detail and again by the larger investigative team. The team also used the Consolidated Criteria for Reporting Qualitative Research (COREQ) 32-item checklist to guide our methods and reporting [55].

### Limitations

Despite these strengths, the results are limited in important ways. For example, our sample size was small and included only nine family physicians. While we did assess data saturation and found no new information after the eighth interview, it is possible that we reached data saturation prematurely by not interviewing participants with sufficient diversity to allow for more variety in responses. For example, although we sampled purposively to ensure that we interviewed rural and urban, community and academic, male and female physicians at varying stages of their professional practice, we were limited to a convenience sample of those who agreed to participate. We also didn’t assess the ordering rates of our sample to engage similar numbers of participants that order images at different rates or actively seek participants in equal numbers who had differing views on imaging conservatively.

## Conclusions

LBP is a common but serious problem that is burdensome and costly for patients and the health system. One of the main drivers of this burden is an overreliance on imaging. Researchers estimate that up to half of all requests for lumbar spine imaging are inappropriate [16–21]. Our study interviewed family physicians in NL to determine context-specific barriers and enablers for reducing unnecessary imaging. We found five key barriers related to seven TDF domains. Successfully changing physician behaviour (which is determined by multiple factors) to reduce inappropriate imaging will require a comprehensive intervention that addresses the most relevant contextual barriers using established behaviour change techniques matched to as many of the implicated domains as possible. The results of our study represent the important first step of this process – identifying the contextual barriers and the domains to which they are related. These results will be used to develop an intervention that will be tested in a later study.

## Supplementary Information

**Additional file 1.**

**Additional file 2.**

## Data Availability

De-identified data collected and analyzed for the current study are available from the corresponding author on reasonable request.

## Abbreviations

LBP: Low back pain
NSLBP: Non-specific low back pain
DI: Diagnostic imaging
CT: Computed tomography
MRI: Magnetic Resonance Imaging
TDF: Theoretic Domains Framework
NL: Newfoundland and Labrador
CAD: Canadian Dollars
SD: Standard deviation
MRC: Medical Research Council
RCT: Randomized controlled trial
BCI: Behaviour change intervention
COREQ: Consolidated criteria for reporting qualitative research
