# Supplementary material for: Barriers to following imaging guidelines for the treatment and management of patients with low-back pain in primary care: a qualitative assessment guided by the Theoretical Domains Framework
Source: BMC Prim Care. 2022 Jun 3;23:143. doi: 10.1186/s12875-022-01751-6 (PMC9164352; doi:10.1186/s12875-022-01751-6)
Supplement: Supplementary file 1 — Additional file 1. [file 12875_2022_1751_MOESM1_ESM.docx]

**Semi-Structured Interview Schedule for 'managing patients with non-specific Low back pain without imaging (X-ray and CT)'.**

**Introduction**: *The general aim of the interview is to help us understand how you make decisions about managing patients with non-specific Low back pain. We want to know what influences your decision to order imaging or not. There are no right or wrong answers; we are trying to understand how different clinicians approach this issue, so please answer frankly.*

I’d like to start with some basic questions about your practice:

1. What type of setting do you practice in? (community vs. Academic; rural versus urban)
2. How many years have you practiced as a family physician?
3. How many FPs in your practice?
4. How many patients in your practice?
5. How hours/day per week are you in Family Practice? What percentage are your patients?
6. Could you please describe the typical process for managing a patient with non-specific low back pain? (prompt: a patient walks in, talk me though what you do) (**Nature of behaviour)**

*Thank you.*

*For the rest of the interview, I have some slightly more specific questions about what influences your use of imaging for patients with non-specific low back pain. Some questions may seem repetitive, but please bear with me as the questions are derived from models of human behaviour and we are trying to figure out which models best apply in this area.*

Are you ready to get started?

Set the scene re: *Keep in mind, then when I ask about managing patient with non-specific back pain, I am referring to those* ***patients without suspected cancer or fracture.*** – I’d like you to think about that for a moment…

**Knowledge**

**(What do they know and how does that influence what they do?)**

1. Are you aware of any guidelines (national, provincial or institutional) about managing patients with non-specific low back pain? What are they?
2. What is your understanding of the guidelines? **(Prompt –what do they say? anything about imaging?)**
3. Do you believe these guidelines to be evidence based? What is your interpretation of the evidence?

**Skills**

1. How much expertise or experience do you think one needs to have to manage back pain without imaging?
2. Are there any skills that are necessary to be considered competent in managing patient with Non-specific low back pain? What are they? (**prompt: what training would someone need if they lack these skills?)**

**Nature of the Behaviour**

1. Is imaging a routine part of managing patients with non-specific low back pain? Why or Why not?
2. Is imaging a part of managing with non-specific low back pain? In what clinical circumstances?

**Memory, Attention and Decision Processes**

1. Is ordering a CT / x-ray an automatic decision or is it something you take time to think about with NS LBP? **(Prompt routine, automatic)**
2. What thought processes guide your decision to use imaging in this NS LBP population? **(Prompt: “What goes through your mind?”)**
3. Is it typically an easy or difficult decision to make? **(Prompt: Weigh pros and cons etc.) - Belief about capabilities regarding the decision.**

**Social/Professional role & identity**

1. If you are monitoring a patient with NS LBP and you don't order a CT or x-ray, do you think you’re doing your job?
2. Is there anything in your professional role as a family physician that influences your decision to use imaging with a NS LBP patient? **(Prompt: professional training, a protocol, an order set, other technologies)**

**Environmental Context & Resources**

1. What aspects of the family practice environment influence your choice of ordering imaging for NS LBP patients? (**Prompt: paper work, ease of access to forms/equipment, internal practice resources)**
2. Are there any resource factors in your community that influence the choice of imaging you use? (**Prompt: personnel, travel, equipment)**
3. Are there any (other) competing tasks or time constraints that might influence whether or not you use imaging for a NS LBP patient?

**Intentions**

1. Do you plan to manage your patients with NS LBP without ordering CT/ x-ray?
2. Do you intend to do so for all NS LBP patients? (**Prompt: Is it case specific?)**

**Goals**

1. How important is it to you to manage patients with NS LBP without imaging? **(Prompt: why or why not? Explain.)**
2. Are there any personal incentives for you to manage patients with NS LBP without imaging? **(Prompt: goals within yourself? External? Is it a priority?)**

**Beliefs about Capabilities**

1. How easy or difficult is it for you personally to manage patients with NS LBP without imaging?
   1. What makes it difficult to manage patients with NS LBP without imaging?
   2. What makes it easy to manage patients with NS LBP without imaging?
2. How easy is it to order the test, physically? Why is it easy?
3. Are you confident that you are able to properly manage a patient with NS LBP without imaging?

**Social Influences**

1. Would any other team members influence whether or not you manage a patient with NS LBP without imaging? **(Prompt: who else; Other clinicians; medical staff including radiologists, nurses, residents, chiropractors; imaging staff or technicians)**
2. Do you ever discuss a case with your colleagues before deciding whether to manage a patient with NS LBP without imaging?
   1. Do your colleagues generally agree with you on this issue?
3. Do your patients with NS LBP and their relatives or care providers influence your care plan? **Prompt: “If patients/family/friends are concerned about either not getting imaging or consequences of imaging, how do you deal with that?”**

**Emotion**

1. How do you feel about managing a patient with NS LBP without imaging? (**Prompt: worry or concern, content, indifferent?)**
2. How do these feelings impact your care plan for that patient?

**Optimism**

1. In general, do you think managing patients with NS LBP without imaging is a good/bad/neutral idea? (**Prompt: is it wasted time, is it valuable, do you care?)**

**Beliefs About Consequences**

1. What do you think would happen if you managed patients with NS LBP with imaging, both positive and negative? **(*prompt: to patients, to colleagues, yourself, short and long term)***
2. What are the drawbacks of managing patients with NS LBP without imaging? **(*prompt: to patients, to colleagues, yourself, short and long term)***
3. What are the benefits of managing patients with NS LBP without imaging? **(*prompt: to patients, to colleagues, yourself, short and long term)***

**Reinforcement**

1. Have you had any experiences (good or bad) in the past around managing patients with NS LBP that influence whether or not you order imaging? **(Prompt: What happened? How did that impact you?)**

**Behavioural Regulation**

1. Do you have any steps or strategies that would encourage you to manage patients with NS LBP without imaging? What are they?
2. In an ideal world, where anything is possible, what strategies would you need in place for managing patients with NS LBP without imaging?
3. If you wanted to implement changes in your own practice (individual/team setting/practice setting) to encourage the management of patients with NS LBP without imaging, what do you think would be the steps necessary to do this?

*That’s all the questions I have for you; has anything occurred to you about this topic that we haven’t asked about?*
