# Supplementary material for: Barriers to following imaging guidelines for the treatment and management of patients with low-back pain in primary care: a qualitative assessment guided by the Theoretical Domains Framework
Source: BMC Prim Care. 2022 Jun 3;23:143. doi: 10.1186/s12875-022-01751-6 (PMC9164352; doi:10.1186/s12875-022-01751-6)
Supplement: Supplementary file 2 — Additional file 2. [file 12875_2022_1751_MOESM2_ESM.docx]

| **Domain** | **Definition, question and sub-constructs** | **Examples** | **Rationale** |
| --- | --- | --- | --- |
| Knowledge | Awareness of the existence of something  **What do they know and how does that influence what they do?**   - Knowledge - Procedural knowledge - Knowledge of task development | The one that I have that I tend to use is the one that the College of Physicians and Surgeons put out. I think the CPSNL has guidelines for low back pain. I’m pretty sure it was CPSNL. | Awareness of guidelines |
|  |  | I did kind of a workshop, a self-directed workshop type of thing. | Clinician reports how they received the guidelines |
|  |  | […your understanding about the guidelines be particularly in relation to the use of imaging?] Don’t image unless there are red flags. | Awareness of what the guidelines report |
|  |  | [Do you believe those guidelines to be evidence based?] Yes. | Awareness that the guidelines are evidence-based |
|  |  | Maybe a feedback mechanism that if you exceed a certain number of routine x-rays, you get a letter or something, like more than the average doctor maybe.  *[Also Behaviour Regulation]* | Knowledge on how they are performing with respect to imaging would help clinician not order imaging. |
|  |  | [What types of things about that conversation do you think are helpful in changing [patients’] perspectives?] …the lack of evidence that there is for doing imaging in this case. | Indicates that the clinician is aware of the evidence around the usefulness of imaging for low back pain. |
| Skills | Ability or proficiency acquired through practice  **What do they know about how they should be doing something and how does that influence whether they do it or not?**   - Skills development - Skills - Competence - Ability - Interpersonal skills - Practice - Skills assessment | [How much experience or expertise do you think one needs to manage back pain without imaging?] You just have to have good education and good clinical skills. | Awareness of the types of skills and training clinicians should have to properly manage patient with low back pain |
|  |  | [Are there any skills that are necessary to be considered competent in managing patients with non-specific low back pain?] Yes. You have to have good clinical skills and if you don’t examine people you are going to miss stuff. I find that is a problem, people image rather than examine.  [Do you mean the history in the physical and neuro examines you talked about before?] Exactly. History physical exam, that basically rules out a lot of the bad stuff. | Awareness of the types of skills and training clinicians should have to properly manage patient with low back pain |
|  |  | [What are the benefits of managing patients without imaging?] I don’t have to order the test in the first place, and my clinical skills are improved because I have to rely on them.  *[Also Beliefs About Consequences]* | Indicates use and development of skills to properly manage patient |
|  |  | [How much experience or expertise do you think one needs to have to manage back pain without imaging?] You just have to have good education and good clinical skills. | Awareness of the types of skills and training a clinician should have to properly manage a patient with low back pain. |
|  |  | [Are there any skills that are necessary to be considered competent in managing patients with non-specific low back pain?] Yes. You have to have good clinical skills and if you don’t examine people you are going to miss stuff. I find that is a problem, people image rather than examine. | Awareness of the types of skills and training a clinician should have to properly manage a patient with low back pain. |
| Social/professional Role and Identity | Coherent set of behaviours and displayed personal qualities of an individual in a social or work setting  **How does who they think they are as a HCP influence whether they do something or not?**   - Professional role - Professional Identity - Identity - Social identity - Professional boundaries - Group identity* (social influence) - Leadership | [If you are monitoring a patient with back pain and you don’t order a CT or x-ray, do you think you’re doing your job?] Yes. | HCP is indicating they don’t need imaging to fulfill the requirements of their job |
|  |  | …I would put the onus on the patient to get better. If they are just sitting in an armchair watching TV and not being active, they can’t expect to get better. | Shifting of responsibility to patient, less responsibility on physician, for managing back pain, larger role to play from patient |
|  |  | [Are there a lot of cases where you feel you can’t convince [patients they don’t need imaging]?] …being an older physician, [patients] tend to believe me. | Indicates HCP believes their identity can sway patient’s beliefs |
| Beliefs about Capabilities | Acceptance of the truth, reality or validity **about an ability, talent or facility** that a person can put to constructive use.  **Do they think they can do what they should do and how does that influence whether they do it or not?**   - Self-confidence - Perceived competence - Self-efficacy - Perceived behavioural control - Self-esteem - Empowerment - Professional confidence - Beliefs | [Are there a lot of cases where you feel you can’t convince [patients that they don’t need imaging]?] Not usually. | Indicates HCP belief in their abilities |
|  |  | [How easy or difficult is it for you personally to manage patients without imaging?] I don’t find it all that difficult. | Indicates HCP belief in their abilities |
|  |  | [Do you feel you are confident in your ability to manage the patient with non-specific low back pain without the imaging?] Yes. | Indicates HCP’s degree of confidence in managing without imaging. |
|  |  | [How do you feel about managing a patient without imaging? Worry, concern, or indifferent that type of thing?] … I’m comfortable with it. | Indicates HCP’s degree of confidence in managing without imaging. |
| Beliefs about Consequences | Acceptance of the truth, reality or validity **about outcomes of a behaviour** in a given situation.  **What are the good and bad things that can happen (or have happened) from what they do and how does that influence whether they’ll do it in the future?**   - Beliefs - Outcome expectancies - Characteristics of outcome expectancies - Anticipated regret* (emotion) - Consequences | 99% of [lower back pain] gets better with self-management. | Belief that if the clinician does nothing and the patient self-manages, they will likely get better |
|  |  | [Would it take a longer then for you to explain to them things or?] It would take longer to explain to them why not to have the imaging than to just order it. Therefore so much inappropriate imaging is ordered, I feel.  *[Also Social Influence]* | Belief that a consequence of not imaging is wasting time. |
|  |  | The competing time constraint would be that it takes longer to explain to them why not to do it. | Indicates that clinician is saying that if they don’t order the image, they have to spend more time explaining to the patient why. |
|  |  | All the effects of radiation on their body, the lack of evidence that there is for doing imaging in this case. The cost is probably the last thing I would mention because they think they deserve everything. I usually try to persuade them on the basis of negative health effects and futility. | Indicates the clinician is aware of the negative consequences associated with imaging. |
|  |  | And the use of resources when other people might need the imaging for something really important. They are butting into a line that just gets longer and longer and makes it more difficult for the appropriate people to access the resource. | Indicates the clinician is aware of the negative consequences associated with imaging. |
|  |  | The patient perceives they are not getting good care [if they aren’t imaged].  *[Also Social Influence]* | Not imaging may result in the patient thinking they aren’t being cared for adequately |
|  |  | [What do you think would happen if you managed patients with imaging?] I would create a lot of paper work, and I would create more time problems for myself reading all the x-rays. It wouldn’t help the patient’s pain and it wouldn’t resolve anything. It would cut more trees down because the hospital always prints 3 or 4 copies of everything, and it’s wasteful, wasteful for all resources and staff. It makes the Radiologist richer. | Indicates the clinician is aware of the negative consequences associated with imaging |
|  |  | [Would you say there are any drawbacks as managing patients without imaging? So say to yourself or colleagues?] Not really. | Indicates perceived lack of negative consequences to not imaging |
|  |  | Occasionally you find an incidental finding that might be a drawback but lots of time incidental findings are incidental, and they don’t need to be managed. Sometimes you get a finding like a dermoid, tumor that shows up in the abdomen, or you have an aneurysm that shows up because it’s calcified, you find spina bifida or something like that. | Indicates aware of negative consequences of not imaging |
|  |  | [What are the benefits of managing patients without imaging?] I don’t have to order the test in the first place, and my clinical skills are improved because I have to rely on them.  *[Also Skills]* | Indicates use and development of skills to properly manage patient |
|  |  | Physicians are not remunerated well in this province so they have to see a lot of people and the faster they do it the better it is for them.  *[Also Environmental Context and Resources]* | Influence of pay incentive on how they practice |
|  |  | It would take longer to explain to them why not to have the imaging than to just order it. | Consequence of not ordering the test (wasting time) |
| Goals | Mental representations of desired outcomes or end states that an individual wants to achieve  **How important is what they do & does that influence whether or not they do it? What standards are they trying to reach, how does that influence whether or not they do it?**   - Goals (distal, proximal) - Goal priority - Goal/target setting - Goals (autonomous/controlled) - Action planning* (behavioural regulation) - Implementation intention | [How important is it to you to manage patients without imaging? That group of patients.] It’s a matter of pride. I want to do the right thing so it’s important to me. | Indicates that the clinician is aware of the negative consequences associated with imaging. |
| Memory, Attention and Decision Making | Ability to retain information, focus selectively on aspects of the environment and choose between two or more alternatives.  **How do their forgetfulness, ability to focus on the behaviour, and/or their decisions about the behaviour influence what they do?**   - Memory - Attention - Attention control - Decision-making - Cognitive overload/tiredness | (Typically an easy or difficult decision to make?) Very easy… Yes, it’s an easy decision not to have testing. | Indicates the ease of making the decision |
|  |  | (Is imaging a routine part of managing patients?) No. | Indicates the automaticity of the decision |
|  |  | [Would there be any circumstances in which you would manage a patient with imaging?]  Yes. If it is a young person and it’s an unusual presentation. Sometimes you find a tumor or something if they have never been imaged before. | This is the decision making process that the clinician undergoes to determine if the patient requires an image. |
|  |  | [Is ordering a CT or an x-ray an automatic decision or is it something you take time to think about with your patient?] No. I think about it all the time. | This is indicating that it’s not an automatic part of their management and they think about that decision with every patient. |
|  |  | I consider imaging if there are some abnormalities in the exam or if I think I need to refer a patient for a specific management, like orthopedics or if I think there is ???? syndrome or something like that, or they have an epidural abscess because they are IV drug user. | This is indicating the things the clinician considers when managing the patient with acute low back pain. |
| Environmental Context and Resources | Any circumstance of a person’s situation or environment that discourages or encourages the development of skills and abilities, independence, social competence, and adaptive behaviour.  **What are the things in their environment that influence what they do and how do they influence what they do?**   - Environmental stressors - Resources/material resources - Organizational culture/climate - Salient events/critical incidents - Person x environment interaction - Barriers and facilitators | [Would you say that’s similar when you’re doing locums for family physicians practices in rural areas?] Yes. All I have to do is right a form and boom, done. | Indicates that locum rural areas are similar to city practices- very easy to order imaging? |
|  |  | (What aspects of a family practice influence your choices of ordering imaging?) Tell the nurse and done. Where I work there are no barriers to imaging. | Indicates the easy access to resources to facilitate ordering |
|  |  | Physicians are not remunerated well in this province so they have to see a lot of people and the faster they do it the better it is for them.  *[Also Beliefs about Consequences]* | Influence of pay structure on how they practice |
|  |  | Sometimes I get them to see the nurse practitioner because it takes the load off me in the ER and she can educate them. | Resources needed to help clinician not order imaging. |
|  |  | [Are there any other competing tasks or time constraints that might influence whether or not you use imaging with your patient?] No, not really. | Indicates that there are not context specific influences. |
|  |  | It’s easier to stick your name on the x-ray form than it is to sit and explain to the patient and examine them. You can see 5 more patients in the time it takes to do that. | Resources needed to help clinician not order imaging. |
| Social Influence | Those interpersonal processes that can cause individuals to change their thoughts, feelings or behaviours  **What do others (HCP, patients, etc.) think of what they do, and how does that influence what they do?**   - Social pressure - Social norms - Group conformity - Social comparisons - Group norms - Social support - Power - Intergroup conflict - Alienation - Group identity* (social professional role and identity) - Modelling | Except when the patient is persistent and you have to use all your mite in order to persuade them not to have imaging. | This is indicating that the patients may have an impact on how the clinician manages the low back pain. |
|  |  | [Would that be a common occurrence do you think?] Yes. “I’m just here for an x-ray doc.” “I’ve just come to get an x-ray on my hip I got back pain.” Patients come with that expectation. | This is indicating that the patients may have an impact on how the clinician manages the low back pain. |
|  |  | Some people are lazy and want a pill for everything. | Indicates patients’ attempts to influence their care/treatment |
|  |  | [Would it take a longer then for you to explain to them things or?] It would take longer to explain to them why not to have the imaging than to just order it. Therefore so much inappropriate imaging is ordered, I feel. | Because the patient is requesting the image. |
|  |  | [Is there anything in your professional role as a physician that influences your decision to use imaging with a non-specific low back pain patient?] Sometimes when there is insurance or when workers compensation is involved that imaging might be appropriate. | Capacity to influence others even when the individual tries to resist. Insurance company may demand the image. |
|  |  | [Are there any things that make it difficult to manage without imaging?] Other physicians ordering all kinds of x-rays all the time makes it more difficult to do the right thing. | This is indicating that other physician’s behaviour of order test may have an impact on how the clinician manages the low back pain. |
| Emotion | Complex reaction pattern, involving experiential, behavioural and physiological elements, by which the individual attempts to deal with a personally significant matter or event  **How do they feel about what they do and how do those feelings influence what they do?**   - Fear - Anxiety - Affect - Stress - Depression - Positive/negative affect - Burn-out | [Are there any other personal incentives for you to manage patients without imaging?]…Gratification, that’s all. | Indicates a motivating influence of emotion |
|  |  | [How important is it to you to manage patients without imaging? That group of patients.] It’s a matter of pride. | Indicates that the clinician has an emotional response to doing the right thing and doing their job correctly. |
|  |  | [How do you feel about managing a patient without imaging? Worry, concern, or indifferent that type of thing?] Not very concerned. |  |
|  |  | I’m not stressed at looking at all the paper work | Indicates that the clinician is aware of the positive consequences associated with not imaging. And one of the consequences is the lack of stress for the physician. |
| Behavioural Regulation | Anything aimed at managing or changing objectively observed or measured actions.  **What strategies would help/have helped them do what they should do?**   - Self-monitoring - Action planning * (Goals) - Breaking habit | [Do you have any steps or strategies that would encourage you to manage patients without imaging and if so what might they be some of the strategies or steps you use.] Patient education. | Indicates that the clinician thinking of strategies for changing behaviour |
|  |  | [What might make it easy to manage patients without imaging?] Maybe a one page little hand out. “Why didn’t I get an x-ray today?” something like that. A little resource sheet and attached to it back care exercise protocol. | One page handout is a resource. Having that resource may modify the clinician behaviour. |
|  |  | Written information for the patient makes it easier. | One page handout is a resource. Having that resource may modify the clinician behaviour. |
|  |  | I would have an ultrasound machine just to scan to see if there are any obvious, you know sometimes ultrasound can be helpful. I might incorporate the use of ultrasound in my practice. [*Also environmental context and resources*] | Resources need to help clinician not order imaging |
|  |  | I might hire a nurse practitioner, an extra nurse, and I would love to have a physiotherapist in the office. I would refer all my patients to a free exercise program that is sponsored by the government instead of paying for CT scans. Put more gyms in the communities and exercise stuff, and personal trainers so that people can learn how to lift and learn how to bend properly, and do their jobs without hurting themselves. [*Also environmental context and resources*] | Resources need to help clinician not order imaging |
| Nature of the Behaviour**  (Not a TDF-2 domain but we are using it in the LBP study) | Description of the behaviour/process.  **What do you do and is that different from what you should do?**   - Routine/automatic habit - Breaking habit - Direct experience/past behaviour - Representation of tasks - Stages of Change Model (pre-contemplation, contemplation, preparation, action and maintenance) | I would just take a history first of all to make sure they are not an IV drug user. | Although initially we weren’t planning on coding Nature of Behaviour it may be beneficial to add that to the coding framework. Since we’re using the TDf-2 there is no “nature of behaviour” domain, so we would need to add it. IF we are unsure about what is it clinicians are actually doing when managing patients with acute non-cancerous low back pain, we may want to know if everyone is doing the same thing or does this behaviour vary. |
|  |  | I look to see if there is a rash on the skin or I also ask about their sexual history if there was anything new going on there that might cause a PID or if there was urinary symptoms. Pretty much every system you need to ask about with it. | Same as above |
|  |  | [Then what would you do next?] If all the answers are no and they don’t have any red flags then I examine them. | Same as above |
|  |  | If it’s radiating and I think it’s sciatica I would ask them to ice it. I would see if there were any gait abnormalities or bending abnormalities. Then I would refer them to physio, most likely I would use physio. Even without radiation of pain I would do a back assessment to see if they had any kind of muscle referred pain and see how they are moving, and see if they have any abnormal gates and probably refer them to physio. Usually I ask them to take a good dose of anti-inflammatory and if they can’t take that then NSAIDs, then Acetaminophen and, heat and ice. | Same as above. |
|  |  | And exercises and I often give stretches. | Description of what the clinician does for the patient. |
| Optimism | The confidence that things will happen for the best or that desired goals will be attained  **How does whether they are optimistic/pessimistic influence what they do?**   - Optimism - Pessimism - Unrealistic Optimism - Identity | [In general do you think managing patients with non-specific low back pain without imaging is a good, bad, or sort of neutral idea?] Good. | This is indicating the clinician’s belief in the behaviour generally. |
|  |  | It wouldn’t help the patient’s pain and it wouldn’t resolve anything. It would cut more trees down because the hospital always prints 3 or 4 copies of everything, and it’s wasteful, wasteful for all resources and staff. |  |
| Reinforcement | Increasing the probability of a response by arranging a dependent relationship, or contingency, between the response and a given stimulus  **How have their experiences (good and bad) of doing it in the past influence whether they will do it?**   - Rewards - Incentives - Punishment - Consequences - Reinforcement - Contingencies - Sanctions | [Have you any experiences, good or bad in the past around managing patients that would influence your decision to image or not.] No I haven’t missed anything major thank goodness | Indicates whether past experience / consequences of doing the behaviour will influence whether they will do the behaviour in the future. |
|  |  | [Are there a lot of cases where you feel you can’t convince them?] Not usually. **Sometimes experience helps** with that [convincing patients they don’t need an image] | Indicates that previous experience in having those conversation have helped the clinician in convincing the patient they do not need an image. |
|  |  | [Are there any other personal incentives for you to manage patients without imaging?] Nope. | Indicates that the clinician is doesn’t require a reinforcement to not image |
|  |  | Physicians are not remunerated well in this province so they have to see a lot of people and the faster they do it the better it is for them. |  |
| Intentions | A conscious decision to perform a behaviour or a resolve to act in a certain way  **How does how inclined they are to do something influence whether they will do it?**   - Stability of intentions - Stages of change model - Transtheoretical model and stages of change | I don’t know if it adds anything to the management, I don’t image. | This is what the clinician does. But it also is an indication that they won’t image either. |
